# Validity of the MED4CHILD tool for assessing adherence to the Mediterranean diet in preschool children

**DOI:** 10.1007/s00431-024-05945-1

**Published:** 2025-01-11

**Authors:** Alicia Larruy-García, Pilar De Miguel-Etayo, Nancy Babio, Katherine Flores-Rojas, Rosaura Picáns-Leis, Carlos Gómez-Martínez, María L. Miguel-Berges, J. Alfredo Martínez, Dolores Corella, M. José de la Torre-Aguilar, Rocío Vázquez-Cobela, Sangeetha Shyam, Belén Pastor-Villaescusa, Diana Paola Córdoba-Rodríguez, Helmut Schröder, María Fernández de la Puente, José Manuel Jurado-Castro, Jiaqi Ni, Santiago Navas-Carretero, Rosaura Leis, Mercedes Gil-Campos, Jordi Salas-Salvadó, Luis A. Moreno

**Affiliations:** 1 Growth, Exercise, Nutrition and Development (GENUD) Research Group, Instituto Agroalimentario de Aragón (IA2), Faculty of Health Sciences, Universidad de Zaragoza, Instituto de Investigación Sanitaria de Aragón (IIS Aragón), 50009 Saragossa, Spain; 2 CIBER, Fisiopatología de La Obesidad y Nutrición (CIBEROBN), Instituto de Salud Carlos III (ISCIII), Madrid, Spain; 3 Universitat Rovira I Virgili, Unitat de Nutrició Humana. Grup ANUT‑DSM, Departament de Bioquimica i Biotecnologia, C/Sant Llorenç, 21, 43201 Reus, Spain; 4 Institut d’Investigació Sanitària Pere Virgili (IISPV), Reus, Spain; 5 Metabolism and Investigation Unit, Reina Sofia University Hospital, Maimónides Institute of Biomedicine Research of Córdoba (IMIBIC), University of Córdoba, 14004 Córdoba, Spain; 6 Instituto de Investigación Sanitaria de Santiago (IDIS), Servicio de Neonatología, Hospital Clínico Universitario de Santiago, Santiago de Compostela, Spain; 7 Department of Nutrition Food Science & Physiology, University of Navarra, 31008 Pamplona, Spain; 8 Departamento de Medicina Preventiva, Facultad de Medicina, Universidad de Valencia, Valencia, Spain; 9 Research Group of Pediatric Nutrition, Instituto de Investigación Sanitaria de Santiago (IDIS), Unidad de Gastroenterología, Hepatología y Nutrición Pediátrica del Hospital Clínico Universitario de Santiago, Santiago de Compostela, Spain; 10 Unit of Investigation in Nutrition, Growth and Human Development of Galicia, University of Santiago de Compostela, Santiago de Compostela, Spain; 11 Present Address: Departamento de Nutrición y Bioquímica, Facultad de Ciencias, Pontificia Universidad Javeriana, 110231 Bogotá, Colombia; 12 Hospital del Mar Medical Research Institute (IMIM), Barcelona, Spain; 13 CIBER de Epidemiologia y Salud Pública (CIBERESP), Madrid, Spain; 14 University of Navarra, Center for Nutrition Research, 31008 Pamplona, Spain; 15 IdisNA, Navarra Institute for Health Research, Pamplona, Spain

**Keywords:** Validation study, Mediterranean pattern, Short screener, Childhood, Dietary intake

## Abstract

Most of the available tools to assess adherence to Mediterranean diet (MedDiet) were constructed for adults, having limited applicability to children and adolescents. The aim of this study is to validate a specific questionnaire to assess adherence to MedDiet in children aged 3 to 6 years (MED4CHILD questionnaire). The validation was performed in a baseline examination of a cohort of children who were recruited in schools in seven cities. Of the total sample of 1509 children, we included 858 children aged 3 to 6 years with complete information on adherence to the MedDiet, food consumption, anthropometry and cardiometabolic characteristics. Adherence to the MedDiet was assessed using an 18-item questionnaire, adapted from validated questionnaire for adults using the Delphi method. Food and beverage consumption was assessed using a validated COME-Kids Food and Beverage Frequency Questionnaire. Anthropometric measurements and cardiometabolic risk factors were assessed using standard methods. Statistical analyses included kappa agreement, ANOVA and linear regression models. Higher MED4CHILD scores were associated with higher intakes of food characteristic of the MedDiet. The MED4CHILD questionnaire showed moderate validity, especially for key items of the Mediterranean diet (kappa ranging from 0.333 to 0.665). Direct significant associations were found between MED4CHILD scores and cardiometabolic profile, including waist circumference (*p*), triglycerides and HOMA-IR (*p* < 0.05). *Conclusion*: The 18-item MED4CHILD questionnaire showed moderate validity for assessing adherence to the MedDiet in children aged 3 to 6 years, in terms of agreement with food and beverage consumption and favourable cardiometabolic profile.

**Table:** 

**What is known**: • *The Mediterranean diet has been linked to a number of health benefits and the assessment of children's adherence to this diet is important for public health and research.* • *The tools available to assess MedDiet compliance are mainly for adults.*
**What is new**: • *The MED4CHILD questionnaire is a simple, inexpensive and rapid tool for assessing MedDiet compliance in children.* • *This tool showed moderate relative validity and a high score was associated with a favourable cardiometabolic profile.*

**Supplementary Information:**

The online version contains supplementary material available at 10.1007/s00431-024-05945-1.

## Introduction

The Mediterranean diet (MedDiet) is considered one of the healthiest dietary patterns [1, 2]. This can be attributed to the composition of MedDiet, which includes fruits, vegetables, nuts, legumes, whole grains, fish, moderate intake of dairy and limited intake of red and processed meats. Notably, olive oil is the primary source of fat and is the most representative food within the MedDiet [3–5]. In recent decades, food consumption in Mediterranean countries has led to a reduction in the proportion of the population adhering to the MedDiet pattern, especially in children [6].

Overweight and obesity are one of the major public health challenges of the twenty-first century [7]. In Europe, the Mediterranean countries have the highest prevalence of overweight and obesity in children 6 to 9 years [8]. A few studies have described an inverse association between the MedDiet pattern and childhood overweight and obesity [9, 10]. In European children, both at baseline and after a 2-year follow-up period, there was an inverse association between adherence to the MedDiet and overweight and obesity [11, 12]. A recent systematic review provided new evidence showing that interventions based on the Mediterranean diet may be effective in improving the cardiometabolic health in children and adolescents [13].

There are several tools available to assess adherence to the MedDiet, based on quantitative or qualitative aspects of the consumption of the different food components of this dietary pattern. Nevertheless, most of these tools were constructed for adults [14–17] and have limited applicability to children and adolescents [12, 18–21]. In children, the most widely used tool is the KIDMED index, which determines the adherence to the MedDiet [21]. Although the KIDMED questionnaire was developed and published 20 years ago, it still does not include many foods and beverages commonly consumed by children today. Despite its widespread use, KIDMED has limitations in assessing children’s adherence to the MedDiet because it does not adjust food group intakes for recommended portion sizes. In addition, it allows users to report daily consumption of vegetables or legumes, but it does not ensure that these quantities are in line with the dietary guidelines. For example, KIDMED only indicates whether a portion of vegetables or legumes is consumed, without specifying if the amount meets the recommended intake for children, potentially leading to an overestimation of adherence.

Therefore, it is necessary to develop and validate a specific and reliable tool to assess adherence to the MedDiet pattern for use in clinical and epidemiological studies. Then, the MED4CHILD addresses this gap by incorporating recommended portion sizes, allowing for a more accurate assessment of adherence to the MedDiet in early childhood. Furthermore, the MED4CHILD questionnaire adjusts for specific quantities, such as the recommended daily intake of vegetables or legumes, providing a more accurate evaluation of adherence. Hence, this study aims to assess the validity of a questionnaire designed to evaluate adherence to MedDiet in Spanish children aged 3 to 6 years (MED4CHILD questionnaire). In this study, we hypothesise that adherence to the MedDiet, as measured by the MED4CHILD questionnaire, will significantly correlate with improved cardiometabolic factors in preschool children. In addition, we will assess whether this adherence is associated with specific cardiometabolic risk factors. The results of this research will not only improve our understanding of the impact of diet on health but will also inform future studies and guide public health initiatives aimed at promoting healthier eating habits in early childhood.

## Materials and methods

### Study design

This is based on data obtained from the CORALS (Childhood Obesity Risk Longitudinal Study) cohort, a prospective ongoing multicentre study in preschool children aged 3 to 6 years at baseline, with an expected 10-year follow-up [22]. The study was registered under ClinicalTrials.gov ID NCT06317883 (https://corals.es/). In this study, we included information from the baseline assessment. Parents or caregivers agreed to participate in the study by signing the informed consent form. The Ethics Committee of each recruitment centre approved the protocol (CEIm Institut d’Investigació Sanitària Pere Virgili 051/2019, CEIC Córdoba Acta 284/Ref. 4155/2019, CEIC Navarra 2019/18, CEIm Hospital Universitario and CEIC Aragón 9/162, Dr. Peset CEIm 9/19, CEIm de la Fundació Unió Catalana d’Hospitals Ref.19/27 and CEIC Santiago-Lugo 2019/131). The study was conducted following the standards of the Declaration of Helsinki.

### Study sample

Participants aged 3–6 years at baseline were recruited between March 2019 and June 2022 (*n* = 1509). Eligible participants for the CORAL cohort were children attending selected schools in seven Spanish cities (Barcelona, Córdoba, Pamplona, Reus, Santiago de Compostela, Valencia and Zaragoza), with caregivers who provided informed consent. Families were excluded if they had difficulty participating, language or comprehension barriers or an unstable housing situation. For the present study, we included children with complete data for the MedDiet adherence questionnaire and the COME-Kids Food and Beverage Frequency Questionnaire (F&B-FQ), BMI and biochemical parameters. A total of 651 participants were excluded due to missing data. Consequently, a total of 858 participants were included in the analysis.

### Dietary assessment

A trained dietician at each site administered the MedDiet adherence assessment questionnaire (MED4CHILD) and the food and COME-Kids F&B-FQ for the previous year, using information provided by parents or caregivers. The COME-Kids F&B-FQ is a 125-item food frequency questionnaire that has been previously validated [23] to assess the usual food and beverage intake in children aged 3 to 11 years. This questionnaire was selected as the reference method because of its reliability in assessing dietary intake in young populations [23] and its alignment with the dietary components assessed by the MED4CHILD questionnaire. The MED4CHILD questionnaire was developed in the context of the MELIPOP pilot study (www.melipop.es, NCT04597281). It is a dietary 18-item questionnaire on MedDiet adherence in children, adapted from a 14-item MedDiet questionnaire that has been validated in an older population [24]. This questionnaire assesses the consumption of typical MedDiet foods as well as additional foods not traditionally included in this dietary pattern. The adaptation process involved several important changes: (a) questions were revised for clarity and relevance to young children’s eating habits; (b) inapplicable questions were removed, and new questions were added to capture dietary behaviours specific to this age group; (c) portion sizes for each question were determined by a panel of nutrition experts to align with dietary guidelines for young children. The modifications were validated using the Delphi method to ensure the relevance and scientific basis of the questionnaire (*Supplemental *Table 1). Each of the 18 items is scored with 1 point, resulting in a score ranging from 0 to 18. A score of 0 points indicates no adherence to the MedDiet, while a score of 18 points indicates the highest adherence to the MedDiet (*Supplemental *Table 2). Adherence was categorised into quartiles to capture a nuanced view of adherence levels.

**Table 1:** General characteristics of the study population, total and by sex

	Total	Boys	Girls	*p*-value
	*n* = 858	*n* = 426	*n* = 432	
Age (years)	4.9 ± 1.0	4.8 ± 1.1	4.9 ± 1.0	0.018
BMI (kg/m^2^)	16.4 ± 2.0	16.2 ± 1.9	16.5 ± 2.2	0.056
BMI z-score^a^	0.4 ± 1.3	0.2 ± 1.2	0.5 ± 1.4	0.028
WC (cm)	51.7 ± 7.2	51.4 ± 6.2	52.0 ± 8.0	0.562
SBP (mm/Hg)	103.9 ± 13.0	104.4 ± 13.2	103.6 ± 12.8	0.504
DBP (mm/Hg)	64.0 ± 12.3	63.9 ± 12.9	64.1 ± 11.6	0.311
Energy intake (Kcal/day)	1819.2 ± 567.2	1873.5 ± 587.2	1765.6 ± 542.1	≤ 0.001
Total cholesterol (mg/dL)*	164.0; 148.0-181.0	163.5; 148.0-180.0	165.0; 148.0-183.0	0.659
HDL-c (mg/dL)*	56.0; 48.0-65.0	56.0; 49.0-66.0	56.0; 48.0-64.1	0.418
LDL-c (mg/dL)*	95.0; 82.0-111.0	95.0; 81.7-109.0	95.0; 82.0-113.0	0.607
Triglycerides (mg/dL)*	53.0; 43.0-66.0	51.0; 41.7-63.0	55.0; 45.0-69.0	≤ 0.001
Glucose (mg/dL)	76.2 ± 13.1	77.5 ± 16.6	74.9 ± 8.0	≤ 0.001
Insulin (microU/mL)	4.3 ± 3.6	3.8 ± 2.7	4.8 ± 4.3	≤ 0.001
HOMA	0.8 ± 0.9	0.7 ± 0.7	0.9 ± 1.3	≤ 0.001

Data are shown as mean ± standard deviation. The statistical significance was set at *p* ≤ 0.05

*BMI* body mass index, *DBP* diastolic blood pressure, *HDL-c* high-density lipoprotein cholesterol, *LDL-c* low-density lipoprotein cholesterol, *HOMA* homeostatic model assessment, *SBP* systolic blood pressure, *WC* waist circumference

*No normal distribution variables are shown as median; P25-P75

^a^According to Cole et al. (2012)

**Table 2:** Food and nutrient intake assessed by the COME-Kids F&B-FQ according to the quartile distribution of the Mediterranean diet score derived from the MED4CHILD questionnaire

	18-item questionnaire of adherence to the Mediterranean diet for children				*p*-value
	1^s^t quartile (0–8 points), *n* = 271	2^nd^ quartile (9–11 points), *n* = 231	3^rd^ quartile, (12–13 points), *n* = 194	4^th^ quartile (14–18), *n* = 162	
Mean	**7.82**	**10.55**	**12.44**	**14.90**	
Foods (g/day)					
Vegetables	57.3 (51.7 − 62.9)	69.6 (62.8 − 76.3)	76.6 (67.6 − 85.5)	98.3 (86.5 − 110.0)	≤ 0.001
Fruits	134.5 (122.2 − 146.9)	169.0 (142.4 − 195.6)	189.3 (170.4 − 208.2)	264.2 (231.9 − 296.5)	≤ 0.001
Olive oil	21.3 (19.4 − 23.1)	21.7 (19.6 − 23.8)	22.5 (20.1 − 24.9)	20.4 (18.3 − 22.4)	0.628
Butter and cream	2.0 (1.6 − 2.4)	2.1 (1.6 − 2.5)	1.4 (1.1 − 1.7)	1.8 (1.2 − 2.3)	0.153
Refined cereals	71.4 (67.0 − 75.8)	76.2 (70.7 − 81.7)	64.6 (60.0 − 69.2)	63.3 (57.5 − 69.2)	≤ 0.001
Fish	29.4 (27.0 − 31.7)	35.3 (32.2 − 38.5)	37.4 (34.3 − 40.6)	41.7 (35.6 − 47.9)	≤ 0.001
Red meat and sausages	25.0 (22.9 − 27.1)	25.7 (23.6 − 27.8)	27.0 (22.4 − 31.6)	24.9 (21.7 − 28.1)	0.758
Nuts	1.2 (1.1 − 1.4)	1.4 (1.21 − 1.54)	1.6 (1.3 − 1.8)	1.8 (1.5 − 2.1)	≤ 0.001
Dairy products	459.7 (429.8 − 489.7)	501.9 (462.2 − 541.7)	502.4 (464.6 − 540.6)	496.8 (453.0 − 540.6)	0.250
Sugared beverages	136.1 (118.3 − 153.8)	115.6 (98.1 − 133.2)	119.7 (95.5 − 143.9)	105.7 (82.6 − 128.7)	0.196
Nutrients					
Carbohydrates (% E)	44.3 (43.6 − 45.0)	43.9 (43.2 − 44.5)	43.6 (42.7 − 44.4)	44.4 (43.4 − 45.4)	0.455
Proteins (% E)	14.4 (14.1 − 14.6)	15.2 (14.9 − 15.5)	15.4 (15.0 − 15.7)	15.58 (15.22 − 15.9)	≤ 0.001
Total fat (% E)	41.3 (40.6 − 42.0)	40.9 (40.2 − 41.7)	41.0 (40.2 − 41.9)	40.0 (39.0 − 41.0)	0.163
Saturated fat (% E)	14.1 (13.8 − 14.3)	14.1 (13.9 − 14.4)	14.0 (13.7 − 14.3)	13.5 (13.1 − 13.9)	0.031
Monounsaturated fat (% E)	17.9 (17.4 − 18.4)	17.9 (17.3 − 18.4)	18.0 (17.3 − 18.6)	17.4 (16.7 − 18.0)	0.538
Polysaturated fat (% E)	6.4 (6.2 − 6.6)	5.9 (5.7 − 6.1)	6.0 (5.8 − 6.2)	6.0 (5.8 − 6.3)	0.007
Cholesterol (mg/dL)	277.1 (267.3 − 287.0)	290.4 (278.4 − 302.4)	284.0 (265.5 − 302.4)	290.1 (268.2 − 311.9)	0.511
Fibre (g)	13.2 (12.7 − 13.7)	14.3 (13.5 − 15.1)	14.5 (138 − 15.2)	17.7 (16.4 − 18.9)	≤ 0.001
Vitamin C (mg)	80.3 (73.7 − 86.8)	94.1 (82.4 − 105.8)	107.5 (95.8 − 119.3)	127.7 (114.5 − 140.8)	≤ 0.001
Vitamin E (mg)	9.2 (8.8 − 9.7)	8.9 (8.4 − 9.4)	9.5 (8.8 − 10.2)	9.7 (9.0 − 10.5)	≤ 0.001
Folic acid (microg)	228 (219 − 238)	250 (236 − 263)	258 (244 − 272)	291 (273 − 310)	0.233
β-Carotene (microg)	1701 (1593 − 1808)	1936 (1807 − 2066)	2183 (1998 − 2367)	2583 (2333 − 2833)	≤ 0.001

Data are expressed as means (95% IC). The statistical significance was set at *p* ≤ 0.05

*E* energy, *the COME-Kids F&B-FQ* the COME-Kids Food and Beverage Frequency Questionnaire

The validation of the MED4CHILD questionnaire was conducted by comparing the results obtained with the MED4CHILD questionnaire for each item with the same scores estimated with the corresponding items of the validated COME-Kids F&B-FQ [23] (*Supplemental *Table 3). This validation was carried out for 17 of the 18 items. Item 11, related to “sofrito”, was excluded from the validation analysis because of the difficulty in accurately estimating the combination of different small portions of vegetables, which was not assessed in the COME-Kids F&B-FQ. In addition, adherence was also categorised into two groups—low (< 10 points) and high (≥ 10 points)—to assess the relative validity between MED4CHILD and COME-Kids F&B-FQ, allowing a focused comparison of higher adherence. Anthropometric variables were measured by trained registered dietitians. Participants wore underwear and no shoes for the measurements. Height was measured using a SECA® 213 precision stadiometer, while weight was measured using a TANITA® 780PMA scale. Waist circumference (WC) was measured with a SECA® 201 tape measure, positioned midway between the last rib and the iliac crest while the participant was standing. The body mass index (BMI) was calculated as weight (kg) divided by height (m^2^). The BMI z-score was calculated according to Cole’s cut-off [25]. Blood pressure (mmHg) was measured twice in the non-dominant arm in a single session, with a 5-min rest between measurements using an automatic sphygmomanometer (Omron® M3 Intellisense HEM-75051-EV; IOMRON Healthcare Europe B.V.) with a cuff adapted to each child. The average of the two measurements was used to calculate systolic (SBP) and diastolic blood pressure (DBP), following international guidelines (European Society of Hypertension and American Heart Association) for standardised BP assessment. Fasting cardiometabolic risk factors including total cholesterol, high-density lipoprotein cholesterol (HDL-C), low-density lipoprotein cholesterol (LDL-C), triglycerides, glucose and insulin were assessed using standard clinical methods, taking into account regular quality control assessments. The homeostatic model assessment-insulin resistance (HOMA-IR) index was calculated [26].

**Table 3:** Assessment of the relative validity of the Mediterranean diet adherence items obtained from the MED4CHILD questionnaire compared to the COME-Kids F&B-FQ

18-item questionnaire of adherence to the Mediterranean diet for children (MED4CHILD)	Serving	Criteria for 1 point	MED4CHILD (%)^a^	COME-Kids F&B-FQ (%)^b^	% of agreement	Agreement (kappa index)	95% CI
1. Does your child use extra-virgin olive oil as the main culinary fat?		Yes	96.3	96.3	2.42	0.416	0.52; 0.63
2. Does your child consume 3 or more tablespoons of olive oil per day (including that used for frying, salads or in meals away from home)?	1 tbsp = 10 mL	Yes	67.7	13.4	1.60	0.123	− 0.08; 0.43
3. Does your child consume 2 or more servings of vegetables per day? During the week, does your child eat any of these servings as raw vegetables or salad?	1 serving = 50–80 g	Yes	44.9	30.2	2.07	0.334	0.41; 0.57
4. Does your child consume 3 or more small fruits per day?	Small fruit = 100 g	Yes	39.9	15.4	2.96	0.338	0.30; 0.63
5. Does your child consume whole grains (bread, cereals, pasta or rice) 3 or more times a week, instead of refined grains?	-	Yes	40.0	18.4	1.92	0.282	0.30; 0.54
6. Does your child consume at least 1 serving per day of fermented milk, plain yogurt or goat’s or sheep’s cheese?	1 commercial portion of fermented milk or yogurt or 25 g of cheese	Yes	59.9	39.0	1.44	0.167	0.17; 0.38
7. Does your child consume 2–3 or more servings a week of legumes?	1 serving = 40 g raw weight	Yes	79.5	79.1	3.57	0.563	0.68; 0.75
8. Does your child consume 3 or more servings a week of fish/seafood?	1 serving = 40–70 g	Yes	60.0	72.0	2.04	0.333	0.42; 0.56
9. Does your child consume at least 3 servings a week of nuts?	1 serving = 15–20 g	Yes	26.1	25.4	4.96	0.665	0.77; 0.82
10. Does your child consume preferably chicken, turkey or rabbit instead of beef, pork, hamburgers or sausage?	-	Yes	82.6	43.1	0.16	0.176	0.04; 0.47
12. Does your child eat red meat, hamburgers, sausages or derivative/processed meat products less than 2 times a week?	-	Yes	57.2	4.8	1.20	0.048	− 0.05; 0.21
13. Does your child eat less than 1 serving per day of butter or cream?	1 serving = 12 g	Yes	85.7	98.0	1.28	0.112	0.08; 0.30
14. Does your child drink less than 2 glasses a week of carbonated and/or sugary beverages (sodas, coke, juices, nectars)?	-	Yes	72.1	85.7	1.59	0.216	0.25; 0.44
15. Does your child consume chips, gummy worms or sweets less than 1 time a week?	-	Yes	44.8	8.9	1.51	0.148	0.03; 0.42
16. Does your child consume dairy desserts such as custard, ice cream, dairy smoothies, petit suisse and vegetable drinks less than 1 time a week?	-	Yes	41.8	5.0	1.35	0.104	− 0.00; 0.33
17. Does your child consume pastries, stuffed cookies, sweets or cakes less than 2 times a week?	-	Yes	45.8	0.8	1.05	0.014	− 0.05; 0.10
18. Does your child consume pre-cooked or ready-to-eat food less than 1 time a week?	-	Yes	71.7	44.9	1.16	0.067	0.00; 0.23
TOTAL score** (**≥ 10 points)		Adherence^c^	68.4	6.8	1.31	0.058	− 0.03; 0.15

The item corresponding to sofrito (a sauce made of tomato, onion and garlic, slowly cooked in olive oil), item 11, was not included because this item was not part of the the COME-Kids F&B-FQ

*the COME-Kids F&B-FQ* the COME-Kids Food and Beverage Frequency Questionnaire, *tbsp* tablespoon

^a^Percentage of participants scoring 1 on the MED4CHILD

^b^Percentage of participants scoring 1 on the COME-Kids F&B-FQ

^c^Considering adherence a score ≥ 10 points

### Statistical analysis

The current analysis was conducted using the CORALS database updated to 20/01/2023. Study variables were assessed to ensure appropriate statistical analyses. Levene’s test was used to assess homogeneity of variance. Baseline characteristics are described as means and standard deviations (SDs) and median (Pc25-Pc75) and compared using the *t*-test. Mean differences in food and nutrient intakes and their 95% confidence intervals (95% CI), as obtained from the COME-Kids F&B-FQ, according to the MED4CHILD quartiles, were analysed using the analysis of variance (ANOVA), and *p* for trend was also calculated. The relative validity of the MED4CHILD was assessed by calculating the % of agreement compared with the MedDiet scores derived from the COME-Kids F&B-FQ (reference method). The kappa index (95% CI) was also calculated and analysed as follows: > 0.8 almost perfect agreement, between 0.61 and ≤ 0.80 substantial agreement, 0.41 to 0.60 moderate agreement, 0.21 to 0.40 fair agreement and ≤ 0.20 weak agreement [27]. A Bland–Altman plot was performed to assess the agreement between the two scores [28]. Linear regression models adjusted for sex and age were fitted to assess the association between MED4CHILD questionnaire and cardiometabolic risk factors. A sensitivity analysis was performed by excluding unhealthy food items identified in the Bland–Altman plot as contributing to the underestimation of Mediterranean diet adherence by MED4CHILD compared to the COME-Kids F&B-FQ. A further analysis was performed comparing the baseline characteristics between including and excluding participants. Statistical significance was fixed at *p* < 0.05. All the statistical analyses were performed using the SPSS® statistical package (version 25.0, SPSS Inc., Chicago, IL, USA).

## Results

### Descriptive characteristics of the study sample

A total of 651 participants were excluded due to missing data for the MED4CHILD questionnaire (*n* = 36), for the COME-Kids F&B-FQ (*n* = 32), for BMI (*n* = 24) and biochemical parameters (*n* = 559). A total of 858 participants were analysed. No significant differences in baseline characteristics between included and excluded participants were shown, except for a higher proportion of less educated individuals among those included.

The body composition and cardiometabolic risk factors of the participants are shown by sex in Table 1. Girls (50.3%) were older and had lower energy intake, as well as higher z-score BMI, glucose concentrations, triglycerides, insulin and HOMA-IR levels compared to boys. Descriptive characteristics of the study population across MED4CHILD score quartiles are shown in *Supplementary *Table 3.

### Mean food groups and nutrient intakes by quartiles of the MED4CHILD questionnaire

Mean food group and nutrient intakes from the COME-Kids F&B-FQ, according to quartile distribution of the MED4CHILD questionnaire, are shown in Table 2*.* Children in the highest quartile of the MED4CHILD score were more likely to have higher intakes of traditional Mediterranean food groups such as vegetables, fruit, fish and nuts (for all *p* ≤ 0.001) and lower intakes of refined cereals (*p* ≤ 0.001). In terms of nutrient intakes, children in the highest quartiles of the MED4CHILD questionnaire were also more likely to have higher intakes of protein (% of total energy intake), dietary fibre, polyunsaturated fatty acids, vitamin C, vitamin E and β-carotene (*p* ≤ 0.001) and lower intakes of saturated fatty acids.

### Relative validity of the MED4CHILD score

Table 3 shows the relative validity of the MedDiet adherence items obtained from the MED4CHILD. Kappa indices in this study ranged from poor agreement (*κ* = 0.014) for pastries, filled biscuits, sweets or cakes to good agreement (*κ* = 0.665) for the consumption of nuts. Key food items in the MedDiet, such as olive oil (*κ* = 0.416), legumes (*κ* = 0.563), fruits (*κ* = 0.338), vegetables (*κ* = 0.334) and fish (*κ* = 0.333), had moderate kappa values. In contrast, red and processed meats (*κ* = 0.048), butter and cream (*κ* = 0.112) and chips or sweets (*κ* = 0.148), among others, showed low kappa values.

Some items showed similar proportions of children consuming them in both the MED4CHILD and the COME-Kids F&B-FQ questionnaires, such as olive oil (96.3% in MED4CHILD and 96.3% in the COME-Kids F&B-FQ) or legumes (79.5% in MED4CHILD and 79.1% in the COME-Kids F&B-FQ). However, some food groups were overestimated in the MED4CHILD questionnaire, such as vegetables (difference of 14.7%), while others were underestimated, such as seafood (difference of 12.0%). For the total score, which was categorised into two groups (adherent ≥ 10 points or non-adherent < 10 points), there was a slight agreement (*κ* = 0.058).

### Associations between the MED4CHILD questionnaire and cardiometabolic risk factors

Table 4 shows the associations between the MED4CHILD questionnaire and cardiometabolic risk factors. Inverse associations were observed between one-unit increase in the MED4CHILD questionnaire and waist circumference (*β* = − 0.012; 95% CI − 0.203, − 0.141), triglycerides (*β* = − 0.079; 95% CI − 0.193, − 0.105) and HOMA (*β* = − 0.018; 95% CI − 0.030, − 0.017). Positive associations were observed between one-unit increase in the MED4CHILD score and HDL-c (*β* = 0.010; 95% CI 0.270, 0.365) and LDL-c (*β* = 0.100; 95% CI 0.407, 2.043).

**Table 4:** Association (*β* coefficients and 95% confidence intervals) between the Mediterranean diet score derived from the MED4CHILD questionnaire and cardiometabolic risk factors

18-item questionnaire of adherence to the Mediterranean diet for children (MED4CHILD) (*n* = 858)			
Dependent variable	*β* coefficient	95% CI	*p*-value
BMI (kg/m^2^)	− 0.047	− 0.086 to 0.015	0.060
WC	− 0.012	− 0.203 to 0.141	≤ 0.001
HDL-c	0.010	− 0.270 to 0.365	≤ 0.001
LDL-c	0.100	0.407 to 2.043	0.009
TG	− 0.079	− 1.193 to − 0.105	≤ 0.001
Total cholesterol	0.090	0.295 to 2.044	0.072
HOMA	− 0.018	− 0.030 to − 0.017	0.035

Regression linear models adjusted by age and sex. The statistical significance was set at *p* ≤ 0.05

*BMI* body mass index, *HDL-c* high-density lipoprotein cholesterol, *HOMA* homeostatic model assessment, *LDL-c* low-density lipoprotein cholesterol, *TG* triglycerides, *WC* waist circumference

The sensitivity analysis after excluding those unhealthy items identified in the Bland–Altman plot potentially contributing to the underestimation of Mediterranean diet adherence by MED4CHILD compared to the COME-Kids F&B-FQ showed no relevant changes in the overall correlation coefficients with cardiometabolic risk variables (data not shown).

**Fig. 1:**
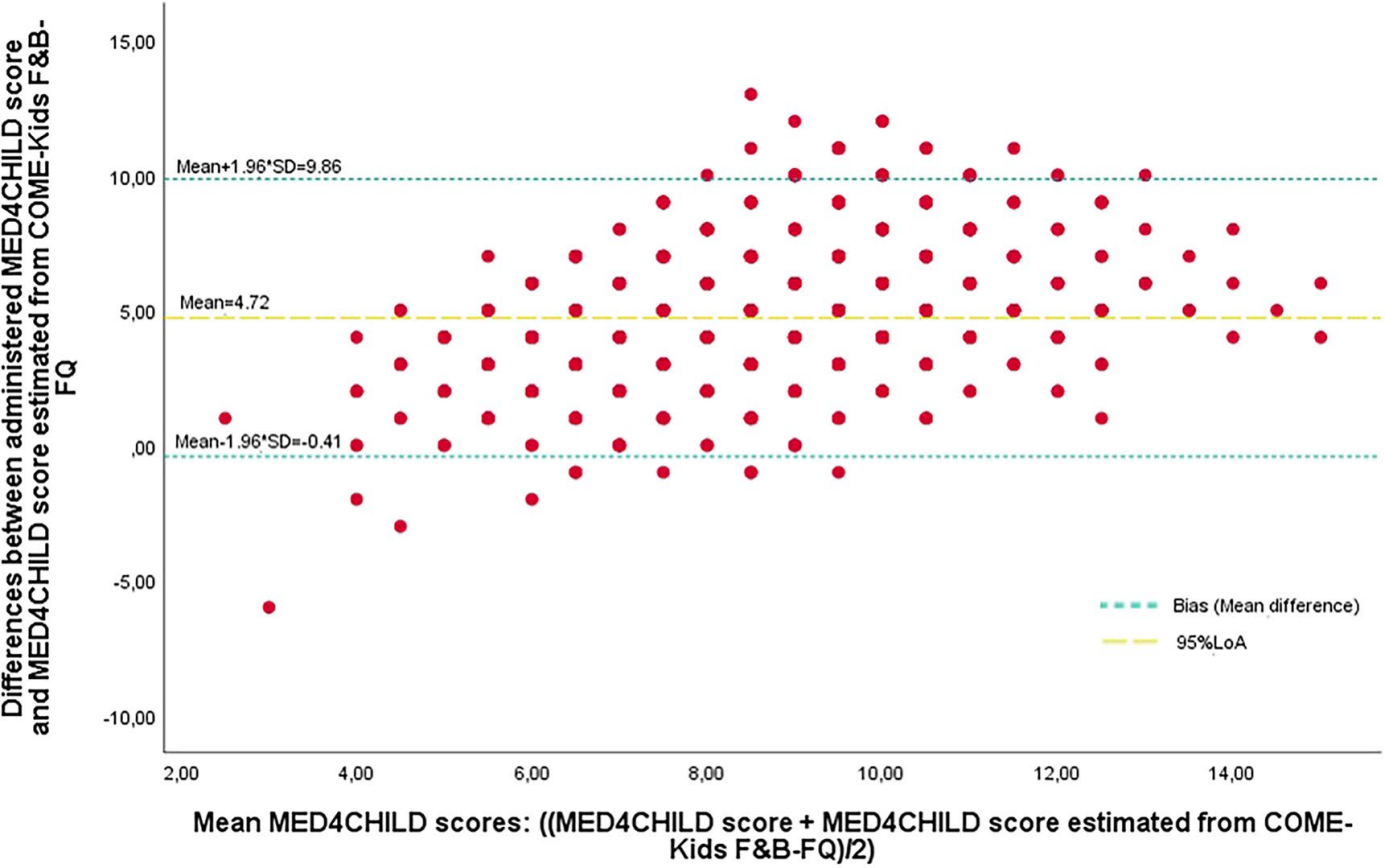
Bland–Altman plot for the agreement between the administered MED4CHILD score and the MED4CHILD estimated from the COME-Kids Food and Beverage Frequency Questionnaire

Figure 1 shows the Bland–Altman plot between adherence to the MED4CHILD questionnaire and the COME-Kids F&B-FQ items. For all levels of MedDiet adherence assessed by MED4CHILD, it underestimated adherence by an average of 5 points, with 95% CIs ranging from 0.41 to 9.44.

## Discussion

The MED4CHILD questionnaire is an easy-to-use, inexpensive and rapid tool designed to assess adherence to the MedDiet in preschool and school-aged children. The MED4CHILD questionnaire has been validated to allow easy assessment of adherence to the MedDiet in clinical and epidemiological settings. Children with higher MED4CHILD scores had higher intakes of key foods and healthier nutrients traditionally consumed in the MedDiet, as assessed by the COME-Kids F&B-FQ. Furthermore, the MED4CHILD score was inversely associated with waist circumference, serum triglyceride concentrations and HOMA and positively associated with HDL-c and LDL-c. Therefore, this validation study supports the use of the 18-item MED4CHILD questionnaire as an adequate tool for assessing adherence to the MedDiet in preschool and schoolchildren aged 3–6 years in Spain.

### MED4CHILD adaptation and validation

The MED4CHILD questionnaire has been adapted for children from the Mediterranean Diet Adherence Screener (MEDAS), a 14-item questionnaire validated in an older population. A previous study using the MEDAS showed strong inverse associations between adherence to the MedDiet and obesity in a population at high cardiovascular risk [24]. Another validated questionnaire used in adults is the energy-restricted MedDiet Adherence Screener (er-MEDAS), which has been validated and shown to detect changes in dietary intake over time and to predict changes in cardiometabolic risk factors [15].

To our knowledge, only the Mediterranean Dietary Serving Score has been validated in the paediatric population [18]. This score is derived from the Mediterranean diet score questionnaire [29], which included participants from a wide age range, from adolescents (aged 12 years) to older adults (aged 83 years). The questionnaire consisted of 14 items that were scored from 0 to 3 points based on the individual’s adherence to the recommendations of the Healthy Eating Pyramid. According to the ROC curve analysis, it showed a significant ability to discriminate between adherents and non-adherents to the Mediterranean dietary pattern, with a sensitivity of 74% and a specificity of 48%.

The KIDMED index is recognised as the most widely used questionnaire to assess the adherence to the MedDiet in children and adolescents [6]. It was originally published in 2004 [21] and has been updated in 2019 [30]. However, the KIDMED questionnaire is based on qualitative information and lacks specific food items tailored to children aged 3 to 6 years. In addition, our understanding of the impact of specific food groups on obesity and related complications has changed in recent years. In particular, the beneficial role of fermented dairy products has been increasingly recognised [31]. To address these limitations, the MED4CHILD questionnaire was developed, to include specific food items commonly consumed by children in this age group and to incorporate new evidence on their associations with obesity and cardiometabolic risk factors. Furthermore, the MED4CHILD score includes a wider range of food items than the KIDMED questionnaire and collects semi-quantitative information, providing a more detailed dietary assessment.

### Nutrient and food group associations

The MED4CHILD showed an association between the high adherence to the MedDiet and high consumption of vegetables, fruit, fish and nuts, which are the main food groups of the MedDiet. However, there was no association with consumption of olive oil or monounsaturated fatty acids. In terms of nutrients, there was also an association between high adherence to the MedDiet and intakes of dietary fibre, vitamins C and E and β-carotene. Adherence to the MedDiet is associated with healthy nutrient intakes and a favourable cardiometabolic risk profile adding scientific data to the well-documented health benefits of the MedDiet. In a validation study carried out in 185 Portuguese adolescents using the KIDMED questionnaire, good agreement with 3-day food recalls was found for the questions on consumption of a second fruit, breakfast, dairy products at breakfast and yogurt or cheese. In our study, the agreement for the consumption of more than one fruit was similar to that obtained in Portuguese adolescents (*κ* = 0.338 vs. *κ* = 0.313); for the consumption of yogurt or cheese, the kappa value we obtained was lower than the kappa obtained in Portuguese adolescents. For olive oil as the main source of fat, we observed moderate agreement (*κ* = 0.416) for the compliance with the recommendations for olive oil consumption, it was *κ* = 0.123; in the Portuguese study, all adolescents reported that the consumption of olive oil was higher than 0 g for at least 1 day of the 3-day dietary record [32]; since in the Portuguese study there were no data to compare with our study because they had only qualitative data, it seems important to include at least semi-quantitative information in the MED4CHILD questionnaire. The agreement between the MED4CHILD items was calculated in comparison with those obtained with the reference method, in this case the full-length and validated COME-Kids F&B-FQ [23]. As in the Portuguese study, we found a slight agreement between the MED4CHILD and the COME-Kids F&B-FQ (*κ* = 0.167 vs. *κ* = 0.058); this difference could be due to the reference method used, 3-day food dietary record vs. COME-Kids F&B-FQ. In addition, it is important to highlight the low kappa coefficients observed for certain items, such as red meat, pastries, sweets and processed food, sugary beverages, butter and cream. This can be explained by the social desirability bias and cognitive load associated with food frequency questionnaires. This bias may lead caregivers to under-report consumption of foods that are perceived as less healthy, thereby affecting recall accuracy for these specific items. Despite this variability, we believe that the overall validity of the MED4CHILD questionnaire is remarkable, as its primary purpose is to capture general dietary quality patterns rather than the precise intake of each individual food. In addition, this questionnaire provides a complementary approach to existing tools by assessing adherence to the Mediterranean diet in children through specific intake quantities aligned with dietary guidelines, offering a practical and child-focused option for research and clinical applications.

Considering the quantitative agreement between the two questionnaires, we found an overestimation when using the MED4CHILD as compared to the COME-Kids F&B-FQ. This finding could be explained by the fact that the COME-Kids F&B-FQ assesses long-term food consumption and the MED4CHILD only assesses semi-quantitative information, reflecting short-term consumption. In addition, discordance may also be due to social desirability bias in reporting less healthy foods; however, sensitivity analyses excluding these items do not significantly affect the associations between MED4CHILD and cardiometabolic risk factors. There are no similar results in children or adolescents; in adults, an average good agreement between the screener and the food frequency questionnaire has been observed [15].

### Adherence to MED4CHILD and cardiometabolic risks

Regarding the cardiometabolic risk factors, adherence to the MED4CHILD questionnaire showed an inverse association with waist circumference measurement, serum triglyceride concentrations and HOMA and a positive association with HDL-c. Some studies also found an inverse association with waist circumference in children [33]. There was also a positive and significant association with serum HDL-C concentration. Despite the lack of association between adherence to the MedDiet and olive oil consumption, we observed high concentrations of HDL-C that could be related to olive oil consumption [34]. These results are supported by previous studies showing that adherence to the MedDiet is positively associated with adiposity markers and cardiovascular parameters in children [35–37]. Unexpectedly, a positive association was observed between adherence to the MedDiet and serum LDL-C concentrations. The unexpected association between higher adherence to the MedDiet and higher LDL-C could be due to several factors including specific dietary components, individual metabolic and genetic differences [38] and potential confounders that were not taken into account as this was not the aim of the study.

This study has some limitations that should be mentioned. First, the use of the COME-Kids F&B-FQ as a reference method. While there are other more robust methods available, such as food diaries, the validated COME-Kids F&B-FQ offers an estimate of long-term dietary intake by accounting for consumption over the last year [39]. However, responses to the COME-Kids F&B-FQ may overestimate dietary intake, particularly for items perceived as healthier indicating that a desirability bias cannot then be excluded. Second, although direct face-to-face interviews were used to improve the accuracy of portion sizes and frequency of consumption, reliance on parent-reported data may still introduce recall bias, especially for less visible foods such as snacks and processed items. Third, another limitation to be acknowledged is the exclusion of the “sofrito” item of the validation due to its complexity and the lack of an equivalent in the reference COME-Kids F&B-FQ, which may affect the accuracy of the assessment of adherence to the MedDiet. Fourth, although minimal baseline differences between included and excluded participants were shown, the slightly higher proportion of less educated individuals among the included cases may influence the generalizability of our findings. Finally, due to the cross-sectional design of the study, causality cannot be inferred from the observed associations between the MED4CHILD score and cardiometabolic risk factors. Future longitudinal studies are needed to establish causal relationships and to further validate the predictive utility of the MED4CHILD tool.

On the other hand, our study also has some strengths, including the large sample size of young Spanish children and the use of cardiometabolic markers, which give us more complete information about the relationship between MedDiet and children’s health. Although several studies have used the KIDMED index to assess adherence to the Mediterranean diet in paediatric populations, there is a notable lack of validation studies for these tools in children, highlighting the added value of MED4CHILD in providing a validated, age-appropriate assessment of dietary adherence.

## Conclusion

In conclusion, the 18-item MED4CHILD questionnaire designed to assess adherence to the MedDiet in children aged 3 to 6 years showed moderate relative validity compared with food and nutrient intake and adherence to the MedDiet calculated using the COME-Kids F&B-FQ. A high MED4CHILD score was also associated with a favourable cardiometabolic profile. The MED4CHILD questionnaire could be used to assess the MedDiet in clinical and epidemiological settings, especially when a rapid assessment is required and to monitor adherence over time. The MED4CHILD tool may not only help to assess dietary quality but also serve as a basis for future longitudinal studies aimed at understanding the relationship between adherence to the MedDiet and the progression of cardiometabolic risk factors in childhood.

## Supplementary Information

Below is the link to the electronic supplementary material.

Supplementary file1 (DOCX 32 KB)

## Data Availability

The datasets generated and analysed during the current study are not publicly available due to data regulations and for ethical reasons, considering that this information might compromise research participants’ acceptance because our participants only gave their consent for the use of their data by the original team of investigators. However, collaborations for data analyses can be requested by sending a letter to the CORALS steering Committee (estudiocoral@corals.es). The request will then be passed to all the members of the CORALS Steering Committee for deliberation.

## Abbreviations

BMI: Body mass index
CI: Confidence intervals
COME-Kids F&B-FQ: COME-Kids Food and Beverage Frequency Questionnaire
DBP: Diastolic blood pressure
HDL-C: High-density lipoprotein cholesterol
HOMA-IR: Homeostatic model assessment-insulin resistance
LDL-C: Low-density lipoprotein cholesterol
MedDiet: Mediterranean diet
SBP: Systolic blood pressure
SDs: Standard deviations
WC: Waist circumference
