# Supplementary material for: Validity of the MED4CHILD tool for assessing adherence to the Mediterranean diet in preschool children
Source: Eur J Pediatr. 2025 Jan 11;184(2):130. doi: 10.1007/s00431-024-05945-1 (PMC11724776; doi:10.1007/s00431-024-05945-1)
Supplement: Supplementary file 1 — Supplementary file1 (DOCX 32 KB) [file 431_2024_5945_MOESM1_ESM.docx]

**Supplementary information**

| **Supplemental Table 1. Modifications performed to create a 18-item questionnaire on MedDiet adherence in children, adapted from a 14-item MedDiet questionnaire** | | | |
| --- | --- | --- | --- |
|  | MEDAS  (14-item questionnaire, adults) | MED4CHILD  (18-item questionnaire, children) | Modification performed |
|  |  |  |  |
| 1 | Do you use olive oil as the principal source of fat cooking? | Does your child use extra-virgin olive oil as the main culinary fat? | Maintained |
| 2 | How much olive oil do you consume per day (including that used in frying, salads, meals eaten away from home, etc.)? | Does your child consume 3 or more tablespoons of olive oil per day (including that used for frying, salads or in meals away from home)? (1 tbsp=10 ml) | Added the frequency of consumption and the serving size |
| 3 | How many servings of vegetables do you consume per day? Count garnish and side servings as ½ point, a full serving is 200 g. | Does your child consume 2 or more servings of vegetables per day?  During the week, Does your child eat any of these servings as raw vegetables or salad? (1 serving= 50-80 g) | Added the frequency of consumption and the serving size was adapted |
| 4 | How many pieces of fruit (including fresh-squeezed juice) do you consume per day? | Does your child consume 3 or more small fruits per day? (Small fruit=100g) | Added the frequency of consumption and the serving size |
| 5 | How many servings of red meat, hamburger or sausages do you consume per day? (full serving is_ 100-150 g.) | Does your child eat red meat, hamburgers, sausages, or derivative/processed meat products less than 2 times a week? | Added the frequency of consumption and some common items to children  Also, the order inside the 18-item questionnaire |
| 6 | How many servings (12g) of butter, margarine or cream do you consumed per day? | Does your child eat less than 1 serving per day of butter or cream? (1 serving=12 g.) | The order inside the 18-item questionnaire |
| 7 | How many carbonated and/or sugar-sweetened beverages do you consumed per day | Does your child drink less than 2 glasses a week of carbonated and/or sugary beverages (sodas, coke, juices, nectars)? | Added the frequency of consumption and some common items to children  Also, the order inside the 18-item questionnaire |
| 8 | Do you drink wine? How much do you consume per week? |  | Deleted |
| 9 | How many servings (150g) of pulses do you consume per week? | Does your child consume 2-3 or more servings a week of legumes? (1 serving= 40 g. raw weight) | Added the frequency of consumption and the serving size was adapted Also, the order inside the 18-item questionnaire |
| 10 | How many servings of fish/seafood do you consume per week (100-150g of fish, 4-5 pieces or 200g of seafood) | Does your child consume 3 or more servings a week of fish/seafood? (1 serving = 40-70 g.) | Added the frequency of consumption and the serving size was adapted Also, the order inside the 18-item questionnaire |
| 11 | How many times do you consume commercial (not homemade) pastry such as cookies or cake per week? | Does your child consume pastries, stuffed cookies, sweets, or cakes less than 2 times a week? | Added the frequency of consumption and some common items to children  Also, the order inside the 18-item questionnaire |
| 12 | How many times do you consume nuts per week (1 serving=30g)? | Does your child consume at least 3 servings a week of nuts? (1 serving = 15-20 g.) | Added the frequency of consumption and the serving size was adapted Also, the order inside the 18-item questionnaire |
| 13 | Do you prefer to eat chicken, turkey or rabbit instead of beef, pork hamburgers or sausages? | Does your child consume preferably chicken, turkey, or rabbit instead of beef, pork, hamburgers, or sausage? | Maintained |
| 14 | How many times per week do you consume boiled vegetables, pasta, rice or other dishes with a sauce of tomato, garlic, onion or leeks saluted in olive oil? | Does your child consume 2 or more times a week cooked vegetables, pasta, rice or other dishes seasoned with tomato, garlic, onion or leek sauce simmered with olive oil (*sofrito*)? | Maintained |
|  |  | Does your child consume chips, gummy worms, sweets less than 1 time a week? | Added due to their consumption in children |
|  |  | Does your child consume whole grains (bread, cereals, pasta, or rice) 3 or more times a week, instead of refined grains? | Added to be more consistent with the mediterranean dietary pattern |
|  |  | Does your child consume at least 1 serving per day of fermented milk, plain yogurt or goat’s or sheep’s cheese? (1 commercial portion of fermented milk or yogurt or 25 g. of cheese) | Added to be more consistent with the mediterranean dietary pattern due to their consumption in children |
|  |  | Does your child consume dairy desserts such as custard, ice cream, dairy smoothies, petit Suisse, vegetable drinks, etc. less than 1 time a week? | Added due to their consumption in children |
|  |  | Does your child consume pre-cooked or ready-to-eat food less than 1 time a week? | Added due to their consumption in children |

| **Supplemental Table 2.** 18-item questionnaire of adherence to the Mediterranean diet in Children | |
| --- | --- |
| **Frequency of consumption** | **Criteria for 1 point** |
| 1. Does your child use extra-virgin olive oil as the main culinary fat? | Yes |
| 1. Does your child consume 3 or more tablespoons of olive oil per day (including that used for frying, salads or in meals away from home)? (1 tbsp=10 ml) | Yes |
| 1. Does your child consume 2 or more servings of vegetables per day? During the week, does your child eat any of these servings as raw vegetables or salad? (1 serving= 50-80 g) | Yes |
| 1. Does your child consume 3 or more small fruits per day? (Small fruit=100g) | Yes |
| 1. Does your child consume whole grains (bread, cereals, pasta, or rice) 3 or more times a week, instead of refined grains? | Yes |
| 1. Does your child consume at least 1 serving per day of fermented milk, plain yogurt or goat’s or sheep’s cheese? (1 commercial portion of fermented milk or yogurt or 25 g. of cheese) | Yes |
| 1. Does your child consume 2-3 or more servings a week of legumes? (1 serving= 40 g. raw weight) | Yes |
| 1. Does your child consume 3 or more servings a week of fish/seafood? (1 serving = 40-70 g.) | Yes |
| 1. Does your child consume at least 3 servings a week of nuts? (1 serving = 15-20 g.) | Yes |
| 1. Does your child consume preferably chicken, turkey, or rabbit instead of beef, pork, hamburgers, or sausage? | Yes |
| 1. Does your child consume 2 or more times a week cooked vegetables, pasta, rice or other dishes seasoned with tomato, garlic, onion or leek sauce simmered with olive oil (*sofrito*)? | Yes |
| 1. Does your child eat red meat, hamburgers, sausages, or derivative/processed meat products less than 2 times a week? | Yes |
| 1. Does your child eat less than 1 serving per day of butter or cream? (1 serving=12 g.) | Yes |
| 1. Does your child drink less than 2 glasses a week of carbonated and/or sugary beverages (sodas, coke, juices, nectars)? | Yes |
| 1. Does your child consume chips, gummy worms, sweets less than 1 time a week? | Yes |
| 1. Does your child consume dairy desserts such as custard, ice cream, dairy smoothies, petit Suisse, vegetable drinks, etc. less than 1 time a week? | Yes |
| 1. Does your child consume pastries, stuffed cookies, sweets, or cakes less than 2 times a week? | Yes |
| 1. Does your child consume pre-cooked or ready-to-eat food less than 1 time a week? | Yes |

**Supplemental Table 3.** Classification of COME-Kids F&B-FQ items according to MED4CHILD questions

|  | **18-item questionnaire of adherence to the Mediterranean diet for children (MED4CHILD)** | **Items from the COME-Kids F&B-FQ** | **Score** |
| --- | --- | --- | --- |
| 1 | Does your child use extra-virgin olive oil as the main culinary fat? | Olive oil (10ml)  Virgin olive oil (10ml)  Pomace olive oil (10ml) | 1 point |
|  |  | Corn oil (10ml)  Sunflower oil (10ml)  Soyabean oil (10ml)  Mixture of the above (10ml) | 0 points |
| 2 | Does your child consume 3 or more tablespoons of olive oil per day (including that used for frying, salads or in meals away from home)? (1tbsp=10 ml) | Olive oil (10ml)  Virgin olive oil (10ml) | ≥2-3 servings per day = 1 point  Less = 0 points |
| 3 | Does your child consume 2 or more servings of vegetables per day? During the week, Does your child eat any of these servings as raw vegetables or salad? (1 serving= 50-80 g) | Vegetables A  Vegetables B  Garlic (1/2 clove) | ≥2-3 servings per day = 1 point  Less = 0 points |
| 4 | Does your child consume 3 or more small fruits per day? (Small fruit=100g) | Citrus fruits  Banana (1 unit)  Fresh fruit (1 portion=100g)  Kiwi (1 serving=100g) | ≥3 servings per day = 1 point  Less = 0 points |
| 5 | Does your child consume whole grains (bread, cereals, pasta, or rice) 3 or more times a week, instead of refined grains? | Black or wholemeal bread (1 slice, 25g)  Wholegrain cereals: muesli, oat flakes, all-bran (30g)  Brown rice (40g uncooked)  Wholemeal pasta (40g uncooked) | ≥3 per week = 1 point  Less = 0 points |
| 6 | Does your child consume at least 1 serving per day of fermented milk, plain yogurt or goat’s or sheep’s cheese? (1 commercial portion of fermented milk or yogurt or 25 g. of cheese) | Plain unsweetened yoghurt (1, 125g)  Fermented milk (100ml)  Other cheeses: cured, semi-cured (manchego, emmental...) (25g)  White or fresh cheese (Burgos, goat's cheese...) (1/2 tub, 30g) | ≥1 serving per day = 1 point  Less = 0 points |
| 7 | Does your child consume 2-3 or more servings a week of legumes? (1 serving= 40 g. raw weight) | Pulses (lentils, beans, chickpeas)  Peas | ≥2-3 servings per week = 1 point  Less = 0 points |
| 8 | Does your child consume 3 or more servings a week of fish/seafood? (1 serving = 40-70 g.) | White fish (1 portion)  Oily fish (1 piece or portion)  Salted fish (1 portion, 30g dry)  Oysters, clams, mussels and similar (3 pcs.)  Squid, octopus, cuttlefish (1 portion, 40-70g)  Crustaceans: shrimps, prawns, crayfish... (2-3 pieces, 30-40g)  Fish by-products (2 pieces)  Fish and shellfish in oil (1/2 tin, 25g) | ≥3 servings per week = 1 point  Less = 0 points |
| 9 | Does your child consume at least 3 servings a week of nuts? (1 serving = 15-20 g.) | Nuts (15-20g) | ≥3 servings per week = 1 point  Less = 0 points |
| 10 | Does your child consume preferably chicken, turkey, or rabbit instead of beef, pork, hamburgers or sausage? | Chicken or turkey with skin (1 portion)  Chicken or turkey without skin (1 portion)  Rabbit (1 portion) | 1 point |
|  |  | Beef or cow meat (1 portion)  Pork (1 portion)  Hamburger (1, 50g) or meatballs (2 pcs)  Bacon (1 slice, 30g) | 0 points |
| 12 | Does your child eat red meat, hamburgers, sausages, or derivative/processed meat products less than 2 times a week? | Beef or cow meat (1 portion)  Serrano or paletilla ham (1 thin slice, 15g)  Ham, cooked ham (1 thin slice, 15g)  Processed meats (15g)  Hamburger (1, 50g) or meatballs (2 pcs) | <1 serving per day=1 point  1 or more per day=0 points |
| 13 | Does your child eat less than 1 serving per day of butter or cream? (1 serving=12 g.) | Cream (1 tablespoon)  Butter (12g) | <1 serving per day=1 point  1 or more per day=0 points |
| 14 | Does your child drink less than 2 glasses a week of carbonated and/or sugary beverages (sodas, coke, juices, nectars)? | Bottled or canned fruit juices (200ml)  Nectars (200ml)  Carbonated beverages with sugar (200ml)  Low calorie carbonated drinks, diet drinks (200ml)  Isotonic drinks (200ml)  Energy drinks (200ml) | <1 serving per day=1 point  1 or more per day=0 points |
| 15 | Does your child consume chips, gummy worms, sweets less than 1 time a week? | Potato crisps chips (50g)  Snacks other than potato crisps (50g)  Sweets, lollipops, jelly beans... (2-3 pcs, 10-15g) | <1 per week=1 point  1 or more per week=0 points |
| 16 | Does your child consume dairy desserts such as custard, ice cream, dairy smoothies, petit Suisse, vegetable drinks, etc. less than 1 time a week? | Milkshakes (200ml)  Petit suisse (55g)  Custard, flan, pudding (1, 130ml)  Ice cream (1 cone)  Vegetable drinks (200ml) | <1 per week=1 point  1 or more per week=0 points |
| 17 | Does your child consume pastries, stuffed cookies, sweets, or cakes less than 2 times a week? | Maria biscuit (3 pcs, 30g)  Wholemeal or fibre biscuits (3 pcs, 30g)  Chocolate biscuits (3 pieces, 30g)  Croissant, ensaimada, sponge cake or other pastries (1 pc, 30g)  Donut (1 pc)  Cupcakes (1 pc)  Cakes (1 piece, 50g)  Churros, porras and similar (1 piece, 20-30g)  Chocolates and pralines (15g)  Mantecados and marzipan (1/2 pc, 45g)  Honey/jam (1 tablespoon, 15g)  Cocoa cream, nocilla or similar (1 dessert spoon, 10g) | <2 per week=1 point  2 or more per week=0 points |
| 18 | Does your child consume pre-cooked or ready-to-eat food less than 1 time a week? | Pizza (60-80g) | <1 per week=1 point  1 or more per week=0 points |

**Abbreviations**: the COME-Kids F&B-FQ, the COME-Kids Food and Beverage frequency questionnaire

**Supplemental Table 4**. General characteristics of the study population according to the 18-item questionnaire of adherence to the Mediterranean diet for children (MED4CHILD).

|  | **18-item questionnaire of adherence to the Mediterranean diet for children (MED4CHILD**) | | | | |
| --- | --- | --- | --- | --- | --- |
|  | **1^st^ quartile**  **7.82 (0-8)**  **n=271** | **2^nd^ quartile**  **10.55 (9-11)**  **n=231** | **3^rd^ quartile**  **12.44 (12-13)**  **n=194** | **4^th^ quartile**  **14.90 (14-18)**  **n=162** | **p for trend** |
| Girls (n, %) | 130 (47.9%) | 116 (50.2%) | 103 (53.1%) | 83 (51.2%) | 0.365 |
| Age (years) | 4.9 (1.0) | 4.9 (1.1) | 4.8 (1.06) | 4.9 (1.1) | 0.788 |
| BMI (kg/m^2^) | 16.6 (2.3) | 16.3 (1.9) | 16.4 (1.89) | 16.2 (1.9) | 0.334 |
| BMI zscore^a^ | 0.5 (1.4) | 0.3 (1.3) | 0.4 (1.28) | 0.2 (1.) | 0.330 |
| WC (cm) | 52.0 (9.4) | 51.6 (5.6) | 52.1 (5.44) | 51.0 (6.9) | 0.469 |
| SBP (mm/Hg) | 105.6 (13.3) | 103.6 (12.3) | 103.3 (13.06) | 102.5 (13.4) | 0.069 |
| DBP (mm/Hg) | 64.5 (12.4) | 64.5 (11.8) | 63.4 (11.59) | 63.4 (13.4) | 0.678 |
| Energy Intake (Kcal/day) | 1825.4 (521.6) | 1843.7 (640.8) | 1787.5 (535.58) | 1811.9 (569.0) | 0.778 |
| Total cholesterol (mg/dL) | 163.2 (27.5) | 166.0 (26.4) | 168.1 (27.54) | 173.7 (59.7) | **0.029** |
| HDL-c (mg/dL) | 56.9 (12.9) | 58.7 (13.9) | 57.6 (12.09) | 56.6 (13.1) | 0.365 |
| LDL-c (mg/dL) | 94.7 (24.4) | 96.2 (23.4) | 100.4 (24.83) | 105.7 (58.2) | **0.005** |
| Triglycerides (mg/dL) | 59.9 (24.5) | 56.8 (24.1) | 55.7 (18.90) | 56.1 (20.1) | 0.160 |
| Glucose (mg/dL) | 77.2 (19.5) | 75.4 (9.0) | 76.0 (9.00) | 75.7 (7.7) | 0.465 |
| Insulin (microU/mL) | 4.4 (3.6) | 4.3 (3.2) | 4.2 (4.57) | 4.3 (2.9) | 0.965 |
| HOMA | 0.9 (0.8) | 0.8 (0.8) | 0.9 (1.48) | 0.8 (0.6) | 0.966 |

Values are presented as means (SD) for continuous variables and n (%) for categorical variables.

a: values were calculated taking into account the IOTF cut-off (1)

**Abbreviations**: BMI: body mass index; WC: waist circumference; SBP: systolic blood pressure; DBP: Diastolic blood pressure; HDL-c: High density lipoprotein cholesterol; LDL-c: Low density lipoprotein cholesterol; HOMA: Homeostatic model assessment
